# ISO 10993-4 Compliant Hemocompatibility Evaluation of Gellan Gum Hybrid Hydrogels for Biomedical Applications

**DOI:** 10.3390/gels10120824

**Published:** 2024-12-13

**Authors:** Mthabisi Talent George Moyo, Terin Adali, Oğuz Han Edebal

**Affiliations:** 1 Department of Medical Biochemistry, Faculty of Medicine, Girne American University, Karmi Campus, Kyrenia 99428, North Cyprus, Turkey; mtgmoyo@gmail.com; 2 Research and Applications Center of Biomedical Sciences, Girne American University, Karmi Campus, Kyrenia 99428, North Cyprus, Turkey; 3 Department of Biomedical Engineering, Faculty of Engineering, Near East University, Nicosia 99138, North Cyprus, Turkey; 4 Clinical Biochemistry Laboratory, Near East University Hospital, Nicosia 99138, North Cyprus, Turkey; oguzhan.edebal@med.neu.edu.tr

**Keywords:** gellan gum, silk fibroin, sodium alginate, hemocompatibility, hydrogels, biomedical applications, ISO 10993-4

## Abstract

This study examines the hemocompatibility of gellan-gum-based hybrid hydrogels, with varying gellan-gum concentrations and constant sodium alginate and silk fibroin concentrations, respectively, in accordance with ISO 10993-4 standards. While previous studies have focused on cytocompatibility, the hemocompatibility of these hydrogels remains underexplored. Hydrogels were formulated with 0.3%, 0.5%, 0.75%, and 1% gellan gum combined with 3% silk fibroin and 4.2% sodium alginate separately, using physical and ionic cross-linking. Swelling behavior was analyzed in phosphate (pH 7.4) and acetic (pH 1.2) buffers and surface morphology was examined by scanning electron microscopy (SEM). Hemocompatibility tests included complete blood count (CBC), coagulation assays, hemolysis index, erythrocyte morphology, and platelet adhesion analysis. Results showed that gellan gum–sodium alginate hydrogels exhibited faster swelling than gellan gum–silk fibroin formulations. SEM indicated smoother surfaces with sodium alginate, while silk fibroin increased roughness, further amplified by higher gellan-gum concentrations. Hemocompatibility assays confirmed normal profiles in formulations with 0.3%, 0.5%, and 0.75% gellan gum, while 1% gellan gum caused significant hemolytic and thrombogenic activity. These findings highlight the excellent hemocompatibility of gellan-gum-based hydrogels, especially the sodium alginate variants, supporting their potential in bioengineering, tissue engineering, and blood-contacting biomedical applications.

## 1. Introduction

The field of biomedical engineering has rapidly advanced, driven by the need for materials that interact harmoniously with the human body, particularly in applications involving direct blood contact [1]. Hemocompatibility, defined as a material’s ability to function without eliciting adverse blood responses, is crucial for any biomaterial used in blood-contacting devices and systems, such as wound dressings, vascular grafts, stents, and drug delivery systems [2,3]. As the demand for these devices grows, so does the need for hemocompatible biomaterials that safely interact with the complex components of blood [4].

Hydrogels have emerged as a promising class of materials for these applications due to their biocompatibility and inherent hemocompatibility, which reduce adverse interactions with blood [5]. Their ability to maintain high water content, mimic soft tissue properties, and form flexible structures makes them suitable for various blood-contacting applications, from vascular graft coatings to drug delivery systems (Figure S1) [6]. Natural biopolymers used in hydrogels, including gellan gum, silk fibroin, and sodium alginate, have attracted significant interest due to their compatibility with biological systems, biodegradability, and low immunogenicity [7,8,9].

Gellan gum, derived from microbial fermentation, offers excellent water absorption, swelling behavior, and gelation properties, making it ideal for blood-contacting applications [10,11,12]. It exhibits minimal hemolysis, reduced platelet adhesion, and a stable coagulation profile—characteristics that render it suitable for blood interaction [13,14,15,16,17]. The gelation mechanism, driven by ionic cross-linking or thermal transition, enables stable gel formation under mild conditions [18,19]. When combined with silk fibroin or sodium alginate, gellan gum forms a hybrid hydrogel that benefits from Silk fibroins’ high mechanical strength and non-thrombogenic properties, enhancing the material’s stability and resistance to mechanical stress [20,21,22,23]. Sodium alginate’s hydrogel-forming ability, low immunogenicity, and biocompatibility complement gellan gum’s properties, resulting in a robust, hemocompatible material with enhanced stability and functionality in blood-contacting applications [24,25,26].

It’s essential to thoroughly assess hydrogels to ensure their full functionality and suitability for biomedical applications [27,28]. Morphological characteristics, including surface roughness and structural features, can significantly influence the hydrogel’s behavior, affecting properties like swelling and overall biocompatibility [28,29]. Additionally, physiochemical properties such as cross-linking density, porosity, and hydrophilicity play a crucial role in how the hydrogel interacts with biological interfaces, influencing various aspects of the biological response [27,29,30]. These characteristics also determine the hydrogel’s stability, degradation rate, and mechanical strength, which are vital for its long-term performance [31,32,33]. To ensure the safety and effectiveness of hydrogels, particularly for blood-contacting applications, adherence to ISO 10993-4 standards is critical [34,35,36,37,38]. These standards provide a framework for evaluating key factors like hemocompatibility and immune responses, helping to minimize risks such as complement activation [34].

Research on gellan gum hydrogels has primarily focused on their gelation properties, mechanical strength, and general biocompatibility [17,18,19,39,40,41,42,43,44,45,46,47,48,49,50]. However, these studies often fall short of meeting the comprehensive hemocompatibility requirements outlined by ISO 10993-4, which includes coagulation, thrombosis, platelet activation, blood cell counts, and immune response (Figure S2) [34,35,36].

Although gellan gum hydrogels, particularly in hybrid formulations with silk fibroin and sodium alginate, demonstrate significant potential for blood-contact biomedical devices, the existing studies barely extend beyond isolated hemocompatibility tests, such as hemolysis. [51,52,53,54]. This is concerning because a full hemocompatibility evaluation, as required by ISO 10993-4, ensures that all relevant interactions with blood components—including effects on coagulation pathways, platelet activation, and immune system responses—are adequately assessed [2,34,37,55,56]. By omitting these critical tests, existing studies fail to capture the complete safety profile of these materials in clinical applications, leading to a gap in the evaluation of their true suitability for blood-contacting devices.

In this study, the hemocompatibility of gellan-gum-based hydrogels for blood-contacting biomedical applications is evaluated in vitro, focusing on their interactions with blood components. Additionally, surface morphology and swelling index are assessed to determine how these properties affect the hydrogels’ behavior and potential performance.

## 2. Results and Discussion

### 2.1. Hydrogel Formulations

The hydrogel formulations, as outlined in Table 1, were synthesized with varying concentrations of gellan gum (0.3%, 0.5%, 0.75%, and 1%), incorporating separate series of silk fibroin (3%) and sodium alginate (4.2%) at fixed concentrations, respectively.

The hydrogels GF1 to GF4 and GA1 to GA4 were designed to investigate the effects of varying gellan-gum concentrations in combination with silk fibroin or sodium alginate. Gellan gum was selected due to its proven hemocompatibility [15,47,53] and its ability to form stable hydrogels under physiological conditions, making it ideal for a wide range of blood-contacting biomedical applications.

The concentrations of gellan gum (0.3% to 1%) were selected based on prior studies demonstrating their ability to provide optimal gelation, mechanical properties, and biocompatibility, all of which are crucial for maintaining structural integrity while performing in biological environments [39,40,41,42,43,45,57].

Silk fibroin was chosen for its non-thrombogenicity and favorable mechanical properties, including tensile strength, elasticity, and biocompatibility [21]. A 3% concentration was selected based on a prior study, which showed that this concentration enhances hydrogel strength and biocompatibility without inducing excessive rigidity [57]. The inclusion of silk fibroin not only improves the mechanical properties of the hydrogel, such as tensile strength and flexibility, but also contributes to a range of tunable properties such as stiffness, swelling behavior, and surface morphology. This variability is particularly beneficial for optimizing the hydrogels for a broad spectrum of blood-contacting biomedical applications, allowing precise control over key characteristics required for specific uses.

In contrast, sodium alginate was used in formulations GA1 to GA4. Known for its ability to form hydrogels through ionic cross-linking, sodium alginate enhances the hydrophilic nature and swelling capacity of the hydrogels [24]. It is also recognized for its favorable hemocompatibility, reducing hemolysis, clotting times, and platelet aggregation, which are vital for blood-contact applications [24,26]. The 4.2% sodium alginate concentration was selected based on prior studies demonstrating that higher sodium alginate concentrations significantly enhance swelling capacity and gel pliability, both of which are critical for applications such as wound dressings [31,58]. Sodium alginate further contributes to the tunability of hydrogel properties by adjusting swelling behavior, stiffness, and surface morphology, making it an essential component for developing hydrogels with tailored characteristics for diverse blood-contacting biomedical applications.

The hydrogels were developed using physical cross-linking and ionic gelation methods to mitigate the drawbacks associated with harsh chemical cross-linking, such as brittleness, potential hemolysis, and the risk of in vitro blood-related complications from residual cross-linking agents, including inflammation, thrombosis, and immune responses.

### 2.2. Swelling Kinetics

Swelling is a critical property that influences a material’s interaction with physiological fluids, affecting surface area, water retention, and mechanical stability. Excessive swelling can compromise structural integrity, leading to degradation or rupture, potentially causing hemolysis or platelet aggregation. Insufficient swelling can reduce bioactivity or impair tissue integration. Thus, evaluating swelling behavior in biologically relevant conditions, such as phosphate buffer (pH 7.4), is essential for understanding how hydrogels behave in a physiological environment. Comparing swelling in acetic buffer (pH 1.2) further assesses material stability under acidic conditions. This helps predict hydrogel performance and ensures biocompatibility in blood-contacting applications.

The swelling ratios of the hydrogels were statistically significant (*p* ≤ 0.05). A Duncan test confirmed significant variations among formulations. Results showed that gellan gum, silk fibroin, sodium alginate concentration, and environmental pH all significantly affect swelling behavior. In both PBS (pH 7.4) and ABS (pH 1.2), hydrogels experienced an initial “shock loss” upon immersion, with greater water release in ABS due to the protonation of carboxyl groups, which weakens ionic interactions. After this phase, swelling occurred due to osmotic pressure and polymer-medium interactions. In PBS, swelling is sustained by repulsive forces between unprotonated carbonyl groups, and prolonged immersion leads to degradation and reduced swelling.

**Figure 1:**
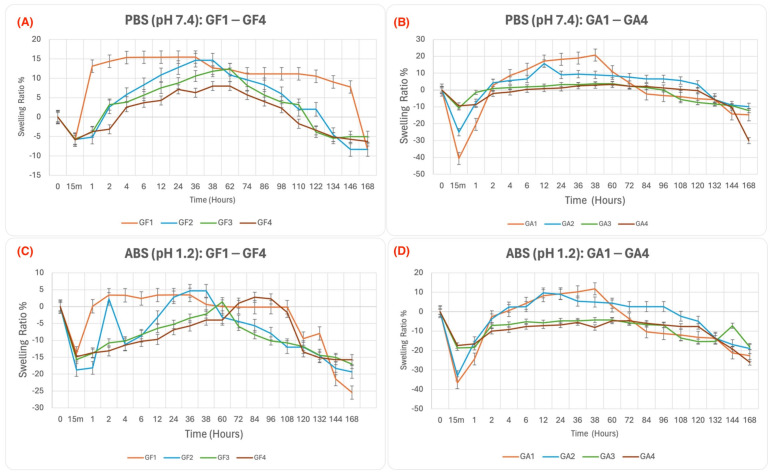
Swelling ratios of gellan gum hydrogels treated with (**A**) silk fibroin in PBS (pH 7.4), (**B**) sodium alginate in PBS (pH 7.4), (**C**) silk fibroin in ABS (pH 1.2), and (**D**) sodium alginate in ABS (pH 1.2). Data are mean ± SD, *n* = 3. Hydrogels in PBS showed significantly higher swelling ratios compared to ABS (*p* ≤ 0.05).

At neutral pH (Figure 1A,B), GA1 (with the lowest gellan-gum concentration) showed the highest degradation (−40.59%), while GF4 (with the highest concentration) showed the least degradation (−5.68%). This suggests that lower gellan-gum concentrations weaken the network, making it more prone to degradation, while higher concentrations provide greater stability. The inclusion of sodium alginate in GA1, which has a high water affinity, may promote disintegration, while silk fibroin in GF4 likely enhances resistance to shock loss. Maximum swelling ratios were higher in hydrogels with sodium alginate, with GA1 showing the highest swelling (20.74% at 38 h) and GA4 the lowest (3.27% at 60 h). Silk-fibroin-based hydrogels showed the highest swelling in GF1 (15.46% at 36 h) and the lowest in GF4 (8% at 38 h). Hydrogels with 0.3% gellan gum (GA1 and GF4) exhibited the highest swelling, with GA1 benefiting from sodium alginate’s presence. After equilibrium, biodegradation caused swelling reduction.

In acidic conditions (Figure 1C,D), hydrogels experienced a greater initial volume reduction due to disrupted hydrogen bonds and protonation of polymer chains. The shock loss was greatest in GA1 (36.56%) and lowest in GF4 (17.42%). Swelling was delayed due to hydrolysis, and hydrogels showed lower peak swelling in ABS than in PBS. In the sodium alginate series, GA1 exhibited the highest peak swelling (11.74% at 38 h), while GA4 showed the lowest (−4.73% at 72 h). In the silk fibroin series, GF1 showed the highest peak swelling (3.46% at 36 h) and GF4 the lowest (2.77% at 84 h). After equilibrium, biodegradation resulted in negative swelling values, with samples degrading more in ABS than in PBS.

Sodium-alginate-based hydrogels exhibited higher swelling ratios than silk-fibroin-based hydrogels in both PBS and ABS, due to alginate’s hydrophilic properties. This is in alignment with previous studies that found similar results [59,60]. Silk fibroin, with its crystalline structure, limits swelling. Sodium alginate forms hydrophilic polymer chains that interact strongly with water, leading to greater swelling. In contrast, silk fibroin’s crystalline regions hinder water absorption.

Swelling behavior provides insight into the potential hemocompatibility of the hydrogels. Hydrogels with higher swelling, like GA1 (sodium-alginate-based), may interact more favorably with physiological fluids, promoting water retention and maintaining structural integrity. Lower swelling hydrogels, like GF4 (silk-fibroin-based), may offer greater resistance to degradation, minimizing adverse interactions with blood. Hydrogels with moderate swelling are likely to be more hemocompatible, balancing water retention and stability. These findings suggest that gellan-gum concentration and environmental pH significantly influence hydrogel swelling behavior, which can predict hemocompatibility. All data are reported as mean ± SD, based on triplicate measurements. These findings suggest that gellan-gum concentration and environmental pH significantly influence the swelling behavior of the hydrogels.

### 2.3. Morphological Analysis

Surface morphology plays a critical role in determining the hemocompatibility of biomaterials, influencing their interaction with blood cells, particularly erythrocytes and platelets, as well as plasma proteins. Previous studies have shown that surface roughness can promote platelet activation and aggregation, potentially leading to thrombus formation and inflammation, which negatively affects hemocompatibility. Conversely, smoother surfaces are typically associated with reduced platelet activation and better blood compatibility.

**Figure 2:**
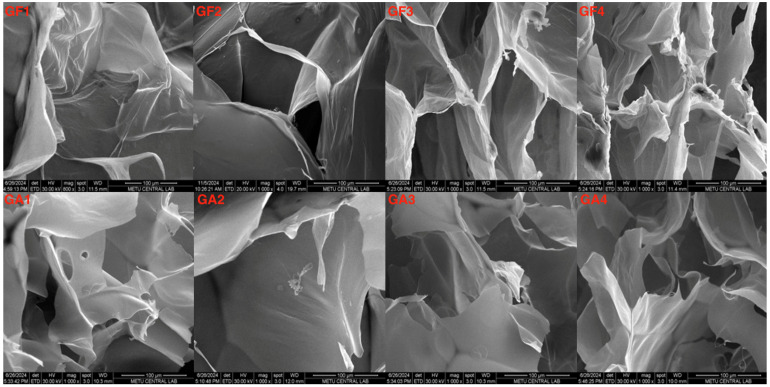
SEM images of scaffold surface morphology at 100 µm magnification, illustrating the surface characteristics of gellan-gum-based hydrogels, with distinct structural features observed across the silk fibroin (**GF1**–**GF4**) and sodium alginate (**GA1**–**GA4**) series, emphasizing the influence of these components on the scaffold surface.

In this study, SEM analysis (Figure 2) revealed distinct differences in the surface characteristics of gellan-gum-based hydrogels, depending on the polymer composition. Gellan gum–sodium alginate hydrogels exhibited smoother surfaces, which are generally beneficial for minimizing unwanted cell adhesion and platelet aggregation. In contrast, gellan gum–silk fibroin hydrogels had rougher surfaces, which could interact differently with blood cells and proteins, potentially leading to greater platelet adhesion and activation.

As the concentration of gellan gum increased, surface roughness also increased. GF1 (0.3% gellan gum + 3% silk fibroin) and GF2 (0.5% gellan gum + 3% silk fibroin) showed moderate roughness, while GF3 (0.75% gellan gum + 3% silk fibroin) and GF4 (1% gellan gum + 3% silk fibroin) exhibited more pronounced roughness, with GF4 showing flakes and folds. These surface irregularities could contribute to higher platelet adhesion, potentially compromising hemocompatibility.

The gellan gum–sodium alginate formulations, on the other hand, maintained smoother surfaces across all concentrations. GA1 (0.3% gellan gum + 4.2% sodium alginate) and GA2 (0.5% gellan gum + 4.2% sodium alginate) displayed the smoothest surfaces, while GA3 (0.75% gellan gum + 4.2% sodium alginate) showed moderate rough-ness. Even GA4 (1% gellan gum + 4.2% sodium alginate), the roughest among the sodium alginate formulations, had a smoother surface than the gellan gum–silk fibroin formulations. These smoother surfaces are likely to reduce platelet activation, supporting their superior hemocompatibility.

The overall smoothness of these scaffolds is associated with reduced thrombogenicity, consistent with prior findings reporting that smoother scaffold surfaces, achieved through optimized fabrication processes, are linked to lower protein adsorption and diminished thrombogenic risk [61,62]

While the primary focus of this study was on hemocompatibility, surface morphology analysis provides valuable insights into the physical characteristics of the hydrogels and their influence on blood-material interactions. Therefore, SEM analysis is essential for understanding the broader implications of material design on biocompatibility and optimizing hydrogels for biomedical use.

### 2.4. Hemocompatibility Analysis Following ISO10993-4 Standards

The hemocompatibility of the gellan-gum-based hydrogels is significantly influenced by their surface characteristics, including roughness and texture. The results from the hemocompatibility assays highlight how these surface properties affect blood interactions, such as coagulation, hemolysis, and erythrocyte morphology.

#### 2.4.1. Hemolysis Index Analysis

The hemolysis index varied among the hydrogel samples, as indicated in Table 2, suggesting that surface characteristics impact red blood cell (RBC) lysis (Table 2). The hemolysis index is a key indicator of hemocompatibility, with values below 30/0 considered acceptable. In this study, the hemolysis indices of gellan-gum-based hydrogels varied based on polymer concentration and composition. The GA1 formulation showed minimal hemolysis (22.7/0), indicating excellent blood compatibility. Similarly, GA2 and GA3 exhibited values of 38.8/1+ and 39/1+, respectively, suggesting normal hemolysis levels that are within an acceptable range for blood-contacting materials. In contrast, increasing gellan-gum concentration to 1% led to higher hemolysis indices, with GA4 showing a value of 39.3/1+. While the hemolysis increased with higher concentrations of gellan gum, the effect was still moderate, indicating that these formulations remain relatively safe for biomedical applications. For the gellan gum–silk fibroin hydrogels, GF1 had a hemolysis index of 46.2/1+, and GF4 had a value of 66.2/1+, both indicating higher hemolysis with increasing gellan-gum concentration.

Overall, formulations with 0.5% and 0.75% gellan gum showed normal hemolysis, while the 1% formulations induced more lysis, though not excessively high. These results suggest that while higher gellan-gum concentrations can increase hemolysis, the overall effect remains mild, making these materials potentially suitable for blood-contact applications.

#### 2.4.2. Coagulation Analysis

The coagulation parameters for the scaffold samples, including the blank and negative control, are summarized in Table 3. Prothrombin time (PT) values ranged from 12.1 to 12.89 s, and activated partial thromboplastin time (aPTT) values ranged from 27.1 to 30.5 s, all within their respective normal ranges, indicating no significant impact on coagulation pathways.

Silk-fibroin-based hydrogels (GF1-GF4) exhibited slightly shorter clotting times compared to sodium-alginate-based formulations (GA1-GA4), suggesting faster activation of the extrinsic pathway. Conversely, sodium alginate hydrogels contributed to longer clotting times, indicating a delay in the intrinsic pathway.

Fibrinogen levels were slightly below the normal range (200–400 mg/dL) across all samples, with sodium alginate formulations (particularly GA4) showing the most notable reduction. However, these deviations did not significantly affect clot formation.

Overall, the hydrogel formulations did not cause significant alterations in coagulation pathways. Silk fibroin hydrogels led to faster clotting, while sodium alginate formulations exhibited slower clotting and reduced fibrinogen levels, suggesting that the hydrogel composition can influence blood clotting behavior in biomedical applications. This assessment is further supported by detailed CBC analyses of blood parameters in treated samples.

#### 2.4.3. Complete Blood Count Evaluation

The CBC analysis for the blank, negative control and hydrogel-treated samples is summarized in Table 4. All key parameters were within normal ranges, indicating minimal impact from the hydrogel treatments. WBC counts ranged from 4.38 to 4.91 × 10³/µL, RBC counts from 5.33 to 5.80 × 10⁶/µL, and HGB levels from 16.0 to 16.4 g/dL, all within standard clinical ranges. Platelet counts were stable across all samples, ranging from 335 to 357 × 10³/µL. MCV and MCH values also remained normal, showing no significant changes in blood cell characteristics or platelet function. Blood reactions induced by biomaterial surfaces remained within the normal range, supporting the CBC findings (Table 4). These results demonstrate the biocompatibility of the hydrogels for blood-contact applications. Such biocompatibility is further validated by the absence of adverse effects on blood cells.

#### 2.4.4. Qualitative Erythrocyte Morphology Analysis and Platelet Adhesion Analysis

**Figure 3:**
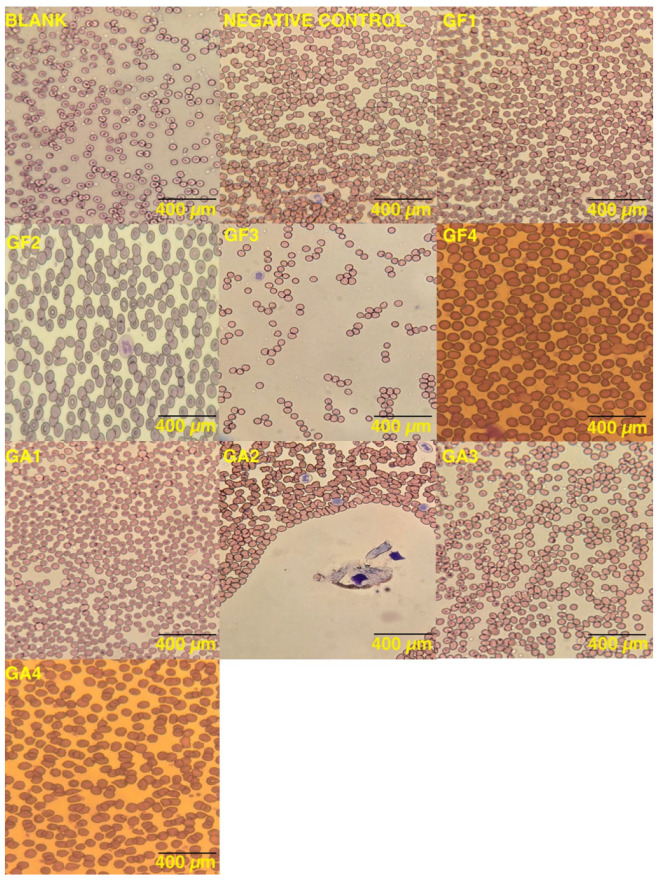
Peripheral blood smears showing erythrocyte morphology and platelet adhesion analysis in blank samples, negative control, and hydrogel-treated blood samples at 400 µm.

Erythrocyte morphology and peripheral blood smear analyses (Figure 3) showed no significant differences in RBC morphology or platelet aggregation between samples with 0.3% and 0.5% gellan gum (GF1, GF2, GA1, and GA2), indicating that the hydrogels did not damage RBCs or induce platelet adhesion. Samples containing 0.75% gellan gum (GF3 and GA3) exhibited minimal hemolysis and a slightly closer, yet normal, platelet distribution. Samples with the highest gellan-gum concentration (GF4 and GA4) showed sparse dysmorphic erythrocytes and some regular platelet aggregation.

Smoother surfaces in GF1, GF2, GA1, and GA2 likely reduced cell membrane stress, maintaining normal erythrocyte morphology. In contrast, GF3 and GA3 showed regular erythrocyte and platelet distribution with no damage. However, GF4 and GA4 displayed sparse dysmorphic erythrocytes and some platelet aggregation. The hemolysis index and SEM micrographs indicated that surface roughness in GF4 contributed to cell lysis and platelet aggregation. Peripheral blood smears were examined under 400× magnification across 10 areas.

The hemocompatibility of the hydrogels is closely linked to their surface characteristics. The rougher surface of GF4 led to a higher hemolysis index, while the smoother surfaces of GF1, GF2, GA1, and GA2 exhibited better blood compatibility. These findings emphasize the importance of optimizing surface smoothness to enhance hemocompatibility for blood-contacting applications, ensuring compliance with ISO 10993-4 standards for safe clinical use.

## 3. Conclusions

This study explored the effects of varying gellan-gum concentrations in combination with silk fibroin and sodium alginate on the swelling behavior, surface morphology, and hemocompatibility of gellan-gum-based hydrogels. The findings demonstrated that sodium-alginate-enhanced hydrogels exhibited superior swelling ratios in both neutral and basic environments, and SEM analysis revealed that surface roughness increased with higher gellan-gum concentrations, which impacted scaffold texture. Hydrogels containing sodium alginate presented smoother surfaces compared to those with silk fibroin.

Hemocompatibility testing revealed that the hydrogels maintained normal coagulation parameters and blood cell counts. Specifically, formulations with lower gellan-gum concentrations exhibited lower hemolytic activity, while higher concentrations showed increased hemolysis and structural rigidity. These results suggest that while the hydrogels are hemocompatible, higher gellan-gum concentrations may lead to undesirable changes in blood compatibility, such as increased hemolytic activity. Future studies should investigate further optimization of hydrogel composition and cross-linking strategies to minimize these effects.

While this study did not directly assess complement activation, indirect indicators, such as hemolysis, suggest potential complement activation, which warrants further investigation. We recommend that future research incorporate specific assays, such as the complement hemolytic assay (CH50) or activation product testing (C3a, C5a), to fully assess the hydrogels’ immunological interactions.

This study highlights the potential of gellan-gum-based hydrogels, particularly those combined with sodium alginate, for applications in biomedical and bioengineering fields, as well as in regenerative medicine. To build on these findings, future studies should explore a wider range of biopolymer concentrations, incorporate additional biomaterials, and conduct in vivo testing to better understand the hydrogels’ performance in complex physiological environments.

In conclusion, this study contributes valuable insights into the design of gellan-gum-based hydrogels for biomedical applications and highlights key areas for future development to optimize hemocompatibility and functionality.

## 4. Materials and Methods

### 4.1. Materials

Silk bombyx mori cocoons were sourced from a local market in North Cyprus, while gellan gum and sodium alginate were procured from Sigma Aldrich (St. Louis, MI, USA). SnakeSkin^®^ dialysis tubing, featuring membranes with a molecular weight cut-off of 3500, was obtained from Thermo Scientific (Waltham, MA, USA). Ethanol (C2H_5_OH), anhydrous sodium carbonate (Na_2_CO_3_), and anhydrous calcium chloride (CaC_l2_) were acquired from EMSURE^®^ Merck chemicals (Darmstadt, Hesse, Germany). Sodium phosphate monobasic (NaH_2_PO_4_), sodium phosphate dibasic (Na_2_HPO_4_), acetic acid (CH_3_COOH), sodium acetate (CH_3_COONa) and pH calibration buffers (pH 4, 7, and 10) for swelling kinetic studies were obtained from Sigma Aldrich (St. Louis, MI, USA). Siemens Thromorel-S was used for the prothrombin time (PT) assays, Siemens Actin-FS for the activated partial thromboplastin time (aPTT) assays, and Siemens Thromboplastin for the fibrinogen assays were obtained from Siemens Healthineers (Erlangen, Bavaria, Germany).

### 4.2. Methods

#### 4.2.1. Extraction and Purification of Silk Fibroin

The authors followed a non-typical method for silk fibroin extraction, based on a validated protocol by Adalı and Uncu, [21] to better control molecular weight and preserve key properties like mechanical strength and biocompatibility, which are crucial for biomedical applications. The extraction process begins with surface cleaning of Bombyx mori cocoons, followed by degumming in 0.1 M sodium carbonate at 75 °C for 2 h, and thorough washing with deionized water. The degummed fibers are then dried overnight. Dissolution of the fibers occurs in a 2:8:1 molar ratio of ethanol, water, and calcium chloride at 75 °C with stirring. The silk fibroin solution is dialyzed using SnakeSkin^®^ (Thermo Scientific, Waltham, MA, USA) tubing in water with periodic changes at 90 rpm for three days and then filtered. This method minimizes degradation, ensuring that silk fibroin retains its functionality for hemocompatibility and tissue engineering. It also aligns with green chemistry principles by utilizing milder reagents, which reduces environmental impact and optimizes the material for blood-contacting applications. Additionally, this approach is reproducible, cost-effective, and practical for broader research and industrial applications.

#### 4.2.2. Sodium Alginate Dissolution

Sodium alginate was prepared at a 4.2% (*w*/*v*) concentration and stirred at 100 rpm and 60 °C until fully dissolved.

#### 4.2.3. Gellan Gum Dissolution

Gellan gum was weighed and dissolved in distilled water to prepare solutions at concentrations of 0.3%, 0.5%, 0.75%, and 1% (*w*/*v*). After stirring at 100 rpm and 90 °C for 2 h until fully dissolved, the solutions were ionically cross-linked by adding 0.03% (*w*/*v*) calcium chloride. The mixture was then stirred for 10 min until homogeneous.

#### 4.2.4. Hydrogel Preparation

Two 10 mL batches of gellan-gum solutions were stirred at room temperature for 2 h at 100 rpm, with one batch containing 1.8 mL of 3% silk fibroin and the other 4 mL of 4.2% sodium alginate. Electrospinning was used to efficiently dissolve and homogenize the solutions, ensuring uniform component distribution. This method enhances control over material properties and facilitates the integration of silk fibroin and sodium alginate into the hydrogel matrix. Samples were stored at −4 °C for 24 h.

#### 4.2.5. Swelling Characteristics

Swelling kinetics of eight hydrogel samples were investigated in 0.1 M phosphate-buffered solution (PBS, pH 7.4) and 0.1 M acetate-buffered saline (ABS, pH 1.2) over a duration of 156 h. Each hydrogel sample was initially weighed (*W_dry_*) and submerged in either PBS or ABS at room temperature. At designated time points, the hydrogels were extracted, gently blotted to remove surface liquid, and promptly reweighed (*W_wet_*). This process was repeated three times to observe swelling behavior over time. The swelling ratio was determined by:

$$SR\left(\%\right)=\left(\frac{Weight\;\left(wet\right)-Weight(Dry)}{Weight(Dry)}\right)\times 100$$

##### Statistical Analysis

Statistical analysis was performed on the swelling kinetic data using Origin 2021 software (OriginLab, Northampton, MA, USA), with significance set at *p* ≤ 0.05. Duncan’s multiple range test and least significant difference (LSD) analysis were applied to evaluate significant differences in swelling ratios among the various hydrogel formulations and between the two buffer environments (PBS and ABS).

#### 4.2.6. Lyophilization

The blend hydrogels underwent simultaneous freeze-drying at −60 °C for 24 h using the lyophilizer chamber of an Alpha 1-2 LSCbasic machine by Martin Christ (Osterode am Harz, Lower Saxony, Germany). This lyophilization process was performed to prepare the samples for morphological, surface, and thermal characterization of the biopolymeric blends.

#### 4.2.7. Scanning Electron Micrography (SEM)

Lyophilized hydrogel scaffolds were prepared for SEM analysis by mounting them onto SEM stubs (Ted Pella, Inc., Redding, CA, USA) with a conductive adhesive. Sputter-coating with gold (SPI Supplied, West Chester, PA, USA) enhanced surface conductivity for imaging. SEM imaging was conducted using a FEI Quanta-series SEM (Thermo Fisher Scientific, Waltham, MA, USA) at 10 kV at the Middle Eastern Technical University Central Laboratory. To visualize microstructure and surface morphology, 100 µm magnifications were used. High vacuum conditions were maintained in the SEM chamber for accurate characterization.

#### 4.2.8. Hemocompatibility Assessment

Blood was collected from a healthy 28-year-old male donor following ethical approval (ethical approval number: YDU/2020/76-955). The samples were labeled and divided into tubes containing sodium citrate (NaCitrate) and dipotassium ethylenediaminetetraacetic acid (K2EDTA) for hemocompatibility assessment (Figure S3). Blank tubes contained untreated blood, while negative controls underwent shaking and incubation. For the plasma coagulation tests, we used 1 mL of plasma and 100 µL of hydrogel. Plasma from Na citrate tubes was tested for PT, aPTT, and fibrinogen after centrifugation at 850 RPM for 10 min. Hydrogel-treated samples were incubated at 37 °C for 1 h before testing for PT, aPTT, and fibrinogen.

For the whole blood count, hemolysis index, and blood smears, we used 2 mL of whole blood and 200 µL of hydrogel. K2EDTA tubes were used for CBC, hemolysis analysis, and peripheral blood smears. Material-contacting blood was used for smears, stained, and analyzed for erythrocyte morphology and platelet adhesion with immersion oil. The hemolysis index was evaluated by centrifuging K2EDTA tubes at 850 RPM for 10 min and analyzing plasma. Hydrogel-treated whole blood was shaken at 100 RPM for 1 h, followed by centrifugation and hemolysis index analysis of the plasma.

All hemocompatibility analyses were performed in a TEMOS-accredited clinical biochemistry laboratory at Near East University Hospital, a recognized research and teaching institution.

##### In-Vitro Coagulation Analysis

PT, aPTT, and fibrinogen were measured using the automated coagulation analyzer SysmexCS-1600–CS-1600 (Sysmex Corporation, Kobe, Hyogo, Japan) and Siemens reagents (Siemens Healthineers, Erlangen, Bavaria, Germany), and results were reported in seconds and mg/dL.

##### Complete Blood Count Analysis

The Sysmex-XN-1000 automated hematology analyzer (Sysmex Corporation, Kobe, Hyogo, Japan) was used to analyze critical parameters such as RBC count, hemoglobin concentration, WBC count with differential, platelet count, and hematological indices like mean corpuscular volume (MCV) and mean corpuscular hemoglobin concentration (MCHC). Regular calibration and quality control checks were performed prior to the experiment to ensure result precision and reliability. CBC data interpretation referenced established ranges, assessing potential hydrogel impacts on blood parameters.

##### Quantitative Hemolysis Index

The hemolysis index analysis was conducted using the Abbott Architect -C4000 automated biochemistry analyzer (Abbott Laboratories, Abbott Park, IL, USA). This involved assessing the degree of hemolysis in the samples, focusing on evaluating the integrity of blood cells and the potential impacts of hydrogels on biochemical measurements.

##### Erythrocyte Morphology and Platelet Adhesion Analysis

Qualitative analysis of erythrocyte morphology and assessment of platelet adhesion was performed to evaluate shape abnormalities in erythrocytes and adherence of platelets following interaction with hydrogel material. Blood samples were shaken at 150 RPM for 45 min at room temperature using an IKA KS 260 shaker, followed by incubation at 37 °C for 1 h. Peripheral blood smears were prepared by fixing specimens on slides with alcohol and staining them using the Wright-Giemsa method. Smears were examined at 400 µm across 10 different areas.

### 4.3. Statistical Evaluation

All results, as shown in Figure 1, were based on triplicate measurements, and reported as averages ± standard deviation (SD). A *p*-value ≤ 0.05 was considered statistically significant using Origin 2021 (OriginLab, Northampton, MA, USA) software. Duncan and least significant difference (LSD) tests were performed to measure the significance among the tested groups and properties.

## Figures and Tables

**Table 1:** Components and ratios of hydrogel formulations.

Sample	Gellan Gum Concentration (%)	Silk Fibroin Concentration (%)	Sodium Alginate Concentration (%)
GF1	0.3%	3%	-
GF2	0.5%	3%	-
GF3	0.75%	3%	-
GF4	1%	3%	-
GA1	0.3%	-	4.2%
GA2	0.5%	-	4.2%
GA3	0.75%	-	4.2%
GA4	1%	-	4.2%

**Table 2:** Quantitative hemolysis index analysis in blank, negative control, and hydrogel-treated blood samples.

Parameter	Standard Range	Blank	Negative Control	GF1	GF2	GF3	GF4	GA1	GA2	GA3	GA4
Hemolysis index	30/0	23/0	28/0	46.2/1+	56.3/1+	59/1+	66.2/1+	22.7/0	38.8/1+	39/1+	39.3/1+

**Table 3:** Coagulation parameters in blank, negative control, and hydrogel-treated samples.

Parameter	Standard Range	Blank	Negative Control	GF1	GF2	GF3	GF4	GA1	GA2	GA3	GA4
PT (Sec)	10–14	12.1	12.5	12.1	12.3	12.4	12.6	12.3	12.5	12.6	12.9
aPTT (Sec)	25–35	28.0	24.9	27.1	27.3	27.5	28.0	28.1	28.3	29.6	30.5
Fibrinogen (mg/dL)	200–400	200.4	172.7	203.1	200.4	205.9	205.9	195.2	195.2	195.2	192.7

**Table 4:** Complete blood count analysis in blank, negative control, and hydrogel-treated samples.

Blood Cells	Measurement Units	Standard Range	Blank Sample	Negative Control	GF1	GF2	GF3	GF4	GA1	GA2	GA3	GA4
White blood cells (WBC)	[10^3^/µL]	4.0–11.0	5.19	5.11	4.62	4.73	4.65	4.91	4.65	4.71	4.38	4.44
Red blood cells (RBC)	[10^6^/µL]	4.2–6.1	5.65	5.61	5.39	5.40	5.50	5.40	5.33	5.42	5.38	5.58
Hemoglobin (HGB)	[g/dL]	12.1–17.2	16.6	16.7	16.1	16.2	16.2	16.1	16.1	16.2	16.0	16.4
Hematocrit (HCT)	[%]	36–54	48.4	48.3	46.5	46.6	47.3	46.7	46.0	46.6	46.4	48.1
Mean Corpuscular Volume (MCV)	[fL]	80–100	85.7	86.1	86.3	86.3	86.0	86.5	86.3	86.0	86.2	86.2
Mean corpuscular hemoglobin (MCH)	[pg]	27–33	29.4	29.8	29.9	30.0	29.5	29.8	30.2	29.7	29.7	29.4
Mean Corpuscular Hemoglobin Concentration (MCHC)	[g/dL]	31.5–35.5	34.3	34.6	34.6	34.8	34.2	34.5	35.0	34.5	34.5	34.1
Platelets (PLT)	[10^3^/µL]	150–450	363	363	338	335	348	348	336	357	365	338
Red Cell Distribution Width-Standard Deviation (RDW-SD)	[fL]	35–55	40.1	40.4	40.8	40.4	40.1	41.0	40.6	40.6	40.5	40.5
Red Cell Distribution Width-Coefficient of Variation (RDW-CV)	[%]	11.5–14.5	12.8	12.9	13.2	13.0	12.9	13.1	13.1	13.1	13.0	13.1
Platelet Distribution Width (PDW)	[fL]	9–17	10.5	10.8	10.4	10.5	11.0	11.1	11.4	10.5	10.7	10.2
Mean Platelet Volume (MPV)	[fL]	7.5–11.5	9.4	9.9	9.5	9.8	9.8	9.7	9.6	10.1	9.9	9.7
Platelet-Large Cell Ratio (P-LCR)	[%]	15–30	20.6	23.3	21.2	22.8	22.7	21.9	21.6	24.0	23.1	21.6
Plateletcrit (PCT)	[%]	0.2–0.5	0.34	0.36	0.32	0.33	0.34	0.34	0.32	0.36	0.36	0.33
Nucleated Red Blood Cells (NRBCs)	[10^3^/µL]	0–2	0.00	0.00	0.00	0.00	0.00	0.00	0.00	0.00	0.00	0.00
Neutrophils (Neut)	[10^3^/µL]	1.8–7.8	2.64	2.72	2.41	2.49	2.41	2.53	2.50	2.51	2.27	2.36
lymphocytes (Lymph)	[10^3^/µL]	1.0–4.8	2.03	1.88	1.71	1.73	1.72	1.60	1.64	1.71	1.64	1.61
Monocytes (Mono)	[10^3^/µL]	0.2–0.8	0.44	0.40	0.40	0.40	0.41	0.45	0.40	0.39	0.37	0.38
Eosinophils (Eo)	[10^3^/µL]	0–6	0.06	0.08	0.08	0.09	0.09	0.10	0.08	0.08	0.08	0.07
Basophils (Ba)	[10^3^/µL]	0–0.2	0.02	0.03	0.02	0.02	0.02	0.03	0.03	0.02	0.02	0.02
Immature Granulocytes (IG)	[10^3^/µL]	0.0–0.1	0.01	0.02	0.01	0.02	0.02	0.02	0.01	0.02	0.01	0.01

## Data Availability

The original contributions presented in the study are included in the article and Supplementary Material, further inquiries can be directed to the corresponding author.

## Supplementary Materials

The following supporting information can be downloaded at https://www.mdpi.com/article/10.3390/gels10120824/s1, Figure S1. Overview of various blood-contacting hydrogel applications in biomedical engineering and regenerative medicine; Figure S2: ISO 10993-4 Parameters; Figure S3: Experimental steps for evaluating the impact of hydrogels on blood components, including CBC analysis, hemolysis index determination, in vitro coagulation parameters (PT, aPTT, and fibrinogen), and examination of peripheral blood smears.
