# Supplementary material for: ISO 10993-4 Compliant Hemocompatibility Evaluation of Gellan Gum Hybrid Hydrogels for Biomedical Applications
Source: Gels. 2024 Dec 13;10(12):824. doi: 10.3390/gels10120824 (PMC11675962; doi:10.3390/gels10120824)
Supplement: Supplementary file 1 [file gels-10-00824-s001.zip › Figure S1.pdf]

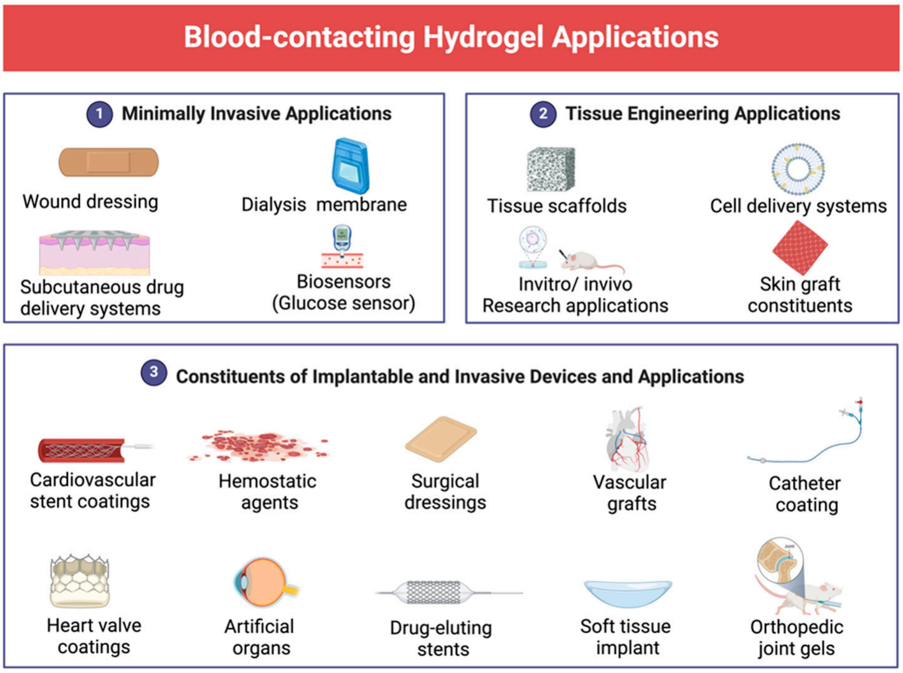

Figure S1. Overview of various blood-contacting hydrogel applications in biomedical engineering and regenerative medicine
