# Supplementary material for: ISO 10993-4 Compliant Hemocompatibility Evaluation of Gellan Gum Hybrid Hydrogels for Biomedical Applications
Source: Gels. 2024 Dec 13;10(12):824. doi: 10.3390/gels10120824 (PMC11675962; doi:10.3390/gels10120824)
Supplement: Supplementary file 1 [file gels-10-00824-s001.zip › Figure S3.pdf]

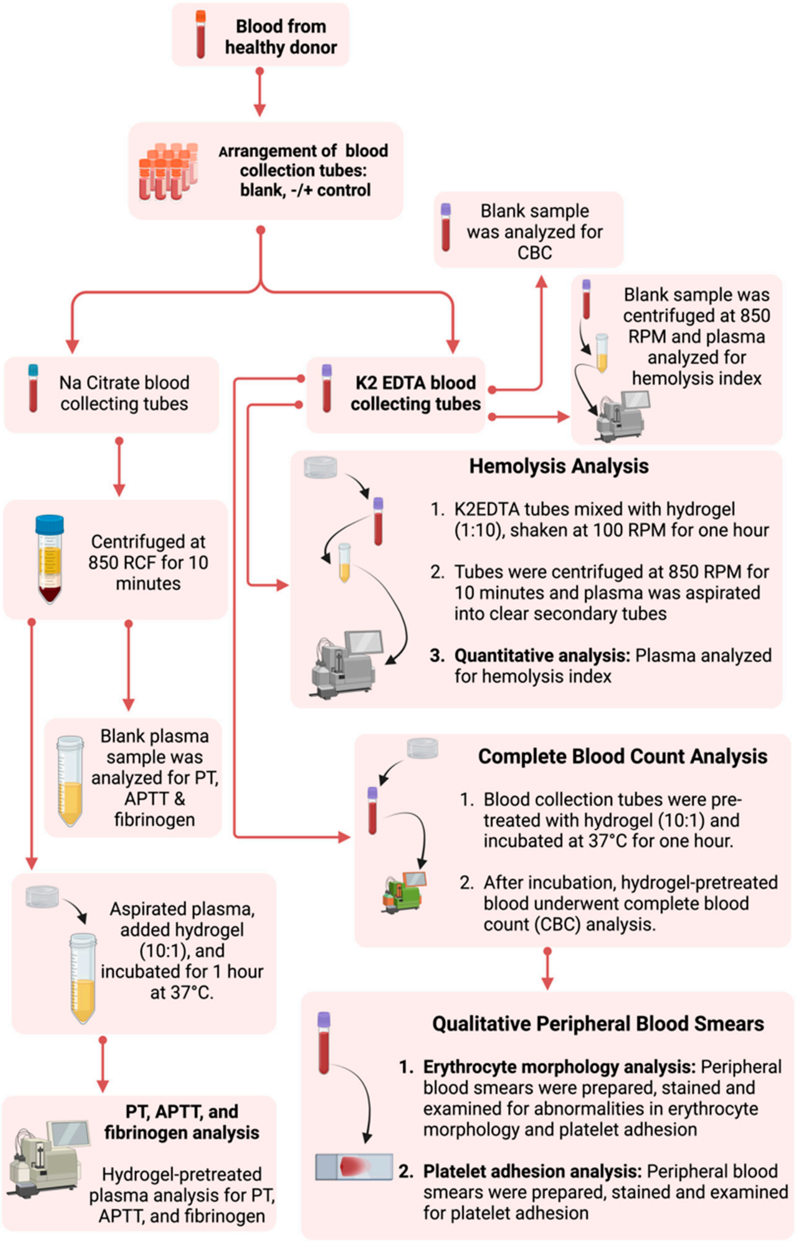

Figure S3. Experimental steps for evaluating the impact of hydrogels on blood components, including CBC analysis, hemolysis index determination, in vitro coagulation parameters (PT, aPTT, and fibrinogen), and examination of peripheral blood smears.
