# Supplementary figures and images for: ISO 10993-4 Compliant Hemocompatibility Evaluation of Gellan Gum Hybrid Hydrogels for Biomedical Applications
Source: Gels. 2024 Dec 13;10(12):824. doi: 10.3390/gels10120824 (PMC11675962; doi:10.3390/gels10120824)

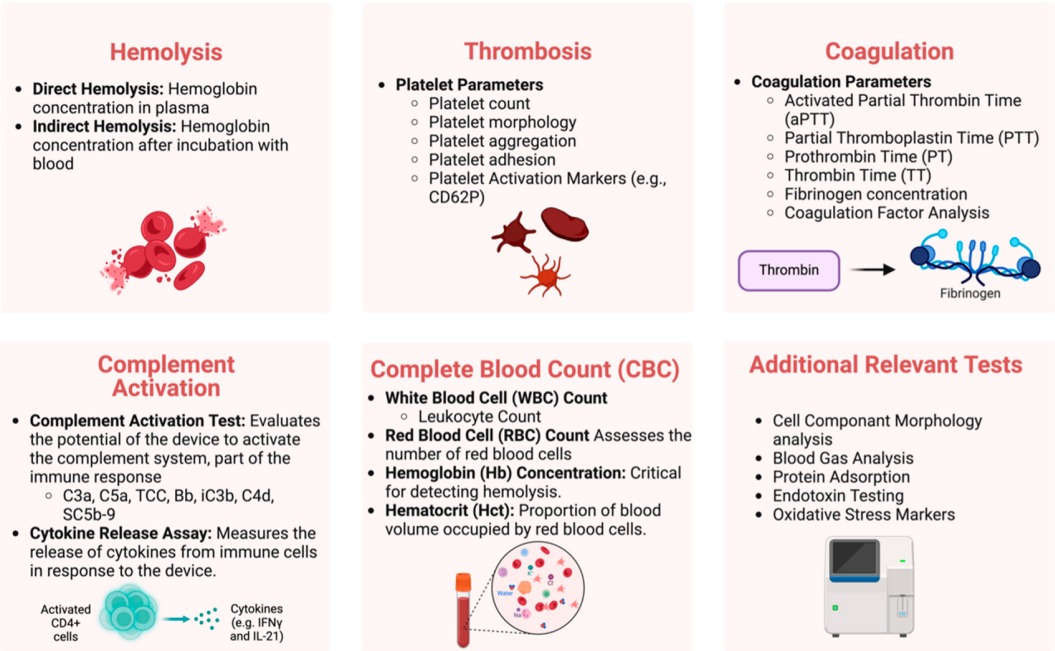

Figure S2 ISO 10993-4 Parameters

Supplement: Supplementary file 1 [file gels-10-00824-s001.zip › Figure S2.pdf]
